# This Wasn’t a Split-Second Decision”: An Empirical Ethical Analysis of Transgender Youth Capacity, Rights, and Authority to Consent to Hormone Therapy

**DOI:** 10.1007/s11673-020-10086-9

**Published:** 2021-01-27

**Authors:** Drew B. A. Clark, Alice Virani

**Affiliations:** 1 Women’s, Gender, and Sexuality Studies, Brandeis University, Rabb 108, Waltham, MA 02453 USA; 2 Provincial Health Services Authority, British Columbia, 4480 Oak Street, Room V2-233, Vancouver, BC V6H 3V4 Canada

**Keywords:** Clinical ethics, Informed consent, Decision-making, Adolescent, Transgender persons, Human rights

## Abstract

Inherent in providing healthcare for youth lie tensions among best interests, decision-making capacity, rights, and legal authority. Transgender (trans) youth experience barriers to needed gender-affirming care, often rooted in ethical and legal issues, such as healthcare provider concerns regarding youth capacity and rights to consent to hormone therapy. Even when decision-making capacity is present, youth may lack the legal authority to give consent. The aims of this paper are therefore to provide an empirical analysis of minor trans youth capacity to consent to hormone therapy and to address the normative question of whether there is ethical justification for granting trans youth the authority to consent to this care. Through qualitative content analysis of interviews with trans youth, parents, and healthcare providers, we found that trans youth demonstrated the understandings and abilities characteristic of the capacity to consent to hormone therapy and that they did consent to hormone therapy with positive outcomes. Employing deontological and consequentialist reasoning and drawing on a foundation of empirical evidence, human rights, and best interests we conclude that granting trans youth with decisional capacity both the right and the legal authority to consent to hormone therapy via the informed consent model of care is ethically justified.

Inherent in providing healthcare for youth lie tensions among best interests, decision-making capacity, rights, and legal authority to make healthcare decisions. While a youth can possess the capacity to make a healthcare decision, they may lack the authority to legally provide consent in some jurisdictions (Salter 2017). Transgender (trans) youth seeking hormone therapy frequently experience barriers to needed care—challenges that are often related to decision-making capacity and legal limitations regarding age of consent. In this analysis, we address the empirical question of whether trans youth can demonstrate the understandings and abilities characteristic of the capacity necessary to make decisions about hormone therapy initiation and the normative question of whether there is ethical justification for granting trans youth the authority to consent to hormone therapy.

## Background

Our research was conducted in British Columbia, Canada, where youth healthcare consent legislation is informed by the mature minor doctrine and generally aligns with the United Nations Convention on the Rights of the Child, which emphasizes evolving capacity, involvement in decision-making, and access to healthcare services as important rights pertaining to the well-being of minors (United Nations 1989). Provincial and territorial mature minor legislation is largely supportive of minor youth authority to consent to healthcare if capacity is demonstrated (Canadian Paediatric Society 2018). The provincial legislation in British Columbia, where our research took place, grants minor youth legal decision-making authority provided they have the capacity to consent to a specific healthcare intervention and that their healthcare provider has determined the intervention to be in their best interests (Infants Act 1996). This approach is similar to practice in the United Kingdom, where youth may consent to treatment based on Gillick competence or the mature minor doctrine (Bird 2011). While the legal landscape in British Columbia differs from that in countries with age-based criteria for consent to medical care, it is important to note that in places such as the United States there exist exceptions to such age of consent laws, allowing minor youth to consent to care related to sexual and reproductive health, mental health, and substance use (National District Attorneys Association 2013).

Healthcare decision-making capacity describes “the degree to which an individual has the ability to understand a proposed therapy or procedure, including its risks, benefits, and alternatives; to communicate relevant questions; and to arrive at a decision consistent with his or her values” (Cummings and Mercurio 2010, 252). Meanwhile, emerging capacity describes the ability that one develops, generally during adolescence, to take on new responsibilities such as healthcare decision-making (Diekema, Mercurio, and Adam 2011). A minor youth may therefore possess capacity to make some healthcare decisions but not others, as the level of capacity necessary to make a healthcare decision is considered proportional to the potential consequences of that decision (Canadian Paediatric Society 2004).

In their cornerstone work, Weithorn and Campbell (1982) used hypothetical case scenarios to study the developmental capacity of adolescents to determine at what age capacity to make decisions about healthcare issues emerges, finding that youth fourteen years of age were as competent decision-makers as adult participants. These findings were confirmed in subsequent studies using hypothetical cases (Scherer and Reppucci 1988) and assessing capacity of youth in clinical settings to make decisions about abortion care (Ambuel and Rappaport 1992) and stimulant medication use for attention deficit/hyperactivity disorder (Greydanus and Patel 1991). In a review paper, Schachter, Kleinman, and Harvey (2011) reached the conclusion that consensus existed in the literature regarding youth capacity to understand information necessary to make medical decisions but that additional empirical research was needed to establish whether adolescents possessed all capacities necessary to consent to medical care. A concern raised in discussions of youth decision-making capacity is that of impulsivity; however, it is important to distinguish between the kinds of decisions that elicit impulsivity versus those that do not (Grootens-Wiegers et al. 2017). Literature examining advances in neuroscience and understanding of adolescent development explains that decisions made in medical contexts are generally not rapid, emotionally charged, or highly subject to peer influence, the conditions that may lead to impulsive decisions in non-medical contexts (Grootens-Weigers et al. 2017; Schwartz et al. 2018). This body of research, legal precedent in multiple jurisdictions, established child rights frameworks, and clinical practice guidelines support the idea that minor youth can possess the developmental capacity to make thoughtful healthcare decisions (Canadian Paediatric Society 2004; Goodlander and Berg 2011; Michaud, Blum, Benaroyo, Zermatten, and Baltag 2015; Weithorn and Campbell 1982).

Healthcare providers are tasked with evaluating whether individuals possess the capacity to make a specific healthcare decision. The British Columbia College of Physicians and Surgeons advises that:

The capacity of a minor is determined by assessing the extent to which the minor’s physical, mental, and emotional development will allow for a full appreciation of the nature and consequences of the proposed treatment, including the refusal of such treatment. (2018, 2)

Assessment of capacity is generally based on understanding of relevant information (e.g., proposed treatment, alternatives), appreciation of this information in the context of one’s own life (e.g., risks and benefits, likely outcomes), reasoning about treatment options, and ability to communicate a clear choice that is consistent with one’s core values (Lo 2013; Palmer and Harmell 2016; Ruhe et al. 2015).

Several articles published in recent years focus on ethical issues arising in clinical practice with trans youth and specifically on the emerging capacity of youth and their legal authority to both access, and consent to, hormone therapy treatment (e.g., Abel 2014; Baltieri, Prado Cortez, and de Andrade 2009; Carroll 2009; Giordano 2007; Shield 2007; Stein 2012; Swann and Herbert 2009). In addition to questions of capacity to consent to hormone therapy, ethical concerns surround determination of youth best interests (e.g., balancing benefits of treatment against potential future harm of fertility implications). However, there is considerable support in the scholarly literature for hormone therapy as a medical intervention for trans youth based on the benefits (e.g. relieving psychological suffering, development of secondary sex characteristics consistent with gender), the relatively low risks of associated harms in adults who access gender-affirming hormone therapy (e.g., thromboembolic disease, erythrocytosis), and the risks of withholding treatment (e.g., suicidality, harassment, violence, use of non-prescription hormones) (Coleman et al. 2012; de Vries et al. 2014; Hembree et al. 2017; Olson, Forbes, and Beltzer 2011; Rosenthal 2014; Shield 2007). While few prospective and longitudinal studies report on outcomes of gender-affirming hormone therapy initiated in adolescence, existing research indicates positive psychosocial outcomes (de Vries et al. 2014) and an absence of clinically significant physiologic outcomes (e.g., lipids, potassium, hemoglobin, prolactin) (Olson-Kennedy et al. 2018). Multiple longitudinal studies on long-term physiological and psychosocial outcomes are currently underway (Olson-Kennedy, Chan, Garofalo, et al. 2019a; Olson-Kennedy, Chan, Rosenthal, et al. 2019b; Trans Youth Can! 2018).

Current standards of care provided by the World Professional Association for Transgender Health (WPATH) recommend that mental health professionals perform a psychodiagnostic and psychiatric evaluation and document their assessment of gender dysphoria, mental health, and eligibility prior to referring youth for hormone therapy (Coleman et al. 2012). Similar recommendations emphasizing the importance of mental health professionals in assessment of gender dysphoria and mental health concerns prior to referral for gender-affirming medical interventions exist for adults (Coleman et al. 2012). This standard model has been challenged in the care of trans adults—due to the focus on pathologizing, in-depth mental health evaluation—with the *informed consent model* emerging as an alternative approach (Ashley 2019; Deutsch 2012; Cavanaugh, Hopwood, and Lambert 2016; Reisner et al. 2015; Schulz, 2018). Within the informed consent model, healthcare providers discuss risks and benefits of treatment options and the potential impact of gender dysphoria on psychosocial well-being in an individual’s sociocultural context, making decisional and mental health supports available but separate from the assessment and informed consent process (Cavanaugh, Hopwood, and Lambert 2016). The informed consent model “seeks to better acknowledge and support patients’ rights of, and their capability for, personal autonomy in choosing care options without the requirement of external evaluation or therapy by mental health professionals” (Cavanaugh, Hopwood, and Lambert 2016, 1149).

The informed consent model has been adopted with positive results and is now supported in the care of adults in the WPATH standards of care (Coleman et al. 2012; Reisner et al. 2015). As Cavanaugh et al. (2016) state, an informed consent model may be appropriate for minor youth, provided that it is used in a developmentally appropriate manner. While capacity assessment is an integral component of assessment with adults and obtaining informed consent from youth is recommended within the standards for care, it is notable that no mention is made of evaluating the decision-making capacity of youth (Coleman et al. 2012). At present, there is a clear divergence in practices concerning hormone therapy assessment for trans individuals, with adults having access to the informed consent model of care, while minor youth are still typically required to undergo potentially pathologizing and burdensome mental health evaluations (Schulz 2018). These differences are reflective of age-based restrictions on the legal authority of minors to consent to hormone therapy (e.g., in the Netherlands and the United States). While capacity-based consent for hormone therapy has been advocated for by multiple legal scholars in recent years (Carroll 2009; Ikuta 2016; Romero and Reingold 2013; Shield 2007), application of the informed consent model of gender-affirming care with minors has received only minimal attention in the literature (Cavanaugh et al. 2016).

This paper is part of a larger qualitative research project, the Trans Youth Hormone Therapy Decision-Making Study (Clark 2018). Consistent with the literature highlighted above, assessment of youth capacity to consent to hormone therapy was raised as an issue of clinical importance by healthcare providers working with trans youth and documented in previous analyses emerging from this study (Clark 2018). We have established that some minor youth with decision-making capacity have provided legal consent for hormone therapy, and we have discussed approaches to clinical ethical decision-making surrounding both youth–parent discordance and shared decision-making (Clark, Marshall, and Saewyc 2020a; Clark, et al. 2020b; Clark, Virani, and Saewyc 2020c). In this paper we seek to expand on these findings, centring broader ethical issues related to youth capacity for decision-making regarding gender-affirming care. Drawing on interviews with trans youth, parents of trans youth, and healthcare providers serving these populations, we addressed the following objectives: to present evidence related to trans youth capacity to consent to hormone therapy and to provide a normative ethical analysis concerning trans youth capacity, rights, and authority to consent to hormone therapy.

## Methods

This research is theoretically grounded in social constructivism and critical realism and follows a gender-affirmative clinical orientation. Ethics board approval for this project was obtained from the University of British Columbia Behavioural Research Ethics Board (H16-01146), University of British Columbia Children’s and Women’s Health Centre of BC Research Ethics Board (H16-01146), the Vancouver Coastal Health Research Institute (V16-2246), and the Northern Health Research Review Committee (RRC H 2016-0042(BLINDED)). The procedures followed were in accordance with the ethical standards of these bodies.

Participants were recruited through healthcare organizations, community organizations, and community events that served trans youth, parents of trans youth, and healthcare providers working with trans youth. The participants included trans youth aged fourteen through eighteen (nineteen being the age of majority in British Columbia) (*n* = 21), parents of trans youth (*n =* 15), and healthcare providers (*n =* 11) who provided hormone therapy readiness assessment/care planning services. Each participant provided informed consent prior to participating in the study. Capacity to provide informed consent was assessed through discussion-based evaluation related to understanding of procedures, risks, and benefits of participation in the study.

Data were collected through one-hour, semi-structured interviews on topics related to making decisions about hormone therapy initiation. Interviews were recorded and later transcribed for analysis with assistance of NVIVO 11 Pro software. Conventional qualitative content analysis was used in analysing participant experiences related to capacity and consent in decision-making about hormone therapy, and directed qualitative content analysis of youth interview data was structured around five core elements of decisional capacity (Hsieh and Shannon 2005). These empirical results informed normative ethical analysis of minor youth capacity and authority to consent to hormone therapy.

## Results

### Perspectives on Capacity and Consent

Healthcare provider, parent, and youth perspectives on capacity and consent related to hormone therapy initiation are presented in the first section of results to provide context for the subsequent analysis of youth capacity to consent to this medical intervention.

#### Healthcare Providers

Healthcare provider concerns surrounding youth capacity to consent for hormone therapy centred around maturity and insight and how these are related to age, development, and mental health. “If a youth doesn’t have the insight yet to kind of understand regret and the potential for regret and the potential for patience as well, it can be really difficult.” Healthcare providers discussed challenges related to youth living with their parents and wanting to transition without disclosure to their parents. “They think a lot about the hormones and what their hormones are going to do to them, but they don’t think a lot about what this actually means, to transition at home without their parents knowing.” In general, healthcare providers were more comfortable with older youth making autonomous decisions about starting hormone therapy.

In evaluating capacity of youth (aged fourteen through eighteen) to consent to hormone therapy, healthcare providers took into account the youth’s understanding of treatment, risks, benefits, and alternatives; insight; coercion; and regret. Most cited British Columbian legislation (Infants Act 1996) as an important framework within their practice. Before supporting initiation of hormone therapy for a particular youth, healthcare providers needed to be certain that the young person had a robust and realistic understanding of hormone therapy, including the range of potential outcomes.

Usually they’re able to demonstrate a broad understanding of what hormones do, and so asking specific questions about: What do you think this is going to do to your body? What are you worried about, if anything? Do you understand these sort of risks that are listed?

Many healthcare providers felt the majority of youth who presented for care were well informed and had the insight necessary to consent to hormone therapy. “Most people, when they come and see us, they’re pretty convinced about what they want in terms of treatment, and so they’ve done their research and they know what they’re looking for.” One participant commented on how the unique lived experiences of trans youth positively impacted their ability to give informed consent.

I actually find most of these kids that have had to ask this question to themselves are way more reflective than their peers of the same age, because they have had to look at questions that other youth may never have had to answer … we see just a breadth of introspection and reflection that is well beyond their years.

Other providers were less confident that youth typically had the capacity to provide informed consent to hormone therapy. One felt that youth were particularly susceptible to coercion or influence around seeking hormone therapy. Tension arose in healthcare provider narratives around fears of youth lacking insight into the potential for regret after starting hormone therapy, which were balanced against supporting access to medically necessary care. “So you’ve got the dilemma of trying to figure out the future and trying to determine whether it’s likely that they will feel good about their decision. They won’t regret it. They won’t feel treated more poorly in society.”

One healthcare provider explicitly spoke about the informed consent model of care, stating that this model was not typically being used with youth and that the standard model of care, with required in-depth mental health assessments, was still the norm in practice. However, this provider had used an informed consent model with youth as young as seventeen. Another participant described using an informed consent approach with youth (without labelling it as such), focusing on capacity, medical screening, safety, and support as the key elements in ensuring that a youth is ready to start hormone therapy. In this healthcare provider’s words, “As long as they understand the information, the same information that somebody who’s twenty-five or forty has to understand around initiating hormone therapy, as long as they understand that, they have a right to receive that healthcare.”

#### Parents

Concerns expressed by parents about youth capacity to make decisions concerning hormone therapy were minimal; however, some parents discussed worries regarding youth insight into the potential for decisional regret in the future. One parent of a youth who started on hormone therapy before the age of fourteen discussed having a higher level of responsibility in the decision-making process than parents of youth who initiated hormone therapy at an older age.

Knowing that at twelve, even at fourteen, developmentally you have a limited ability to process really the full impact of that. And he may say to me one day: “What the fuck, Mom? Why’d you let me?” I don’t think so, but it’s possible.

Another area that elicited questions about youth capacity was their decision-making related to possible long-term fertility implications of hormone therapy, with some parents concerned youth could not know their future fertility intentions. “But, you know, I didn’t want kids at fifteen. I didn’t want kids at twenty. That’s just something that happens later in life.” In contrast, other parents were satisfied that their children had engaged in mature reflection and informed decision-making when coming to conclusions that having biological children was not the right choice for them. “That was a very mature thing for her to come to the conclusion of, and it’s her body, and it’s her future.”

A parent of an older youth discussed her child’s emerging capacity and how her approach to parenting included supporting her child to develop the capacity to make healthcare decisions. Even though she disagreed with the decision to start hormone therapy, she supported her child in making an autonomous decision to start hormone therapy.

And that was almost like my test, you know, as a parent. It’s like I’ve been regurgitating this stuff to you your whole life, that at some point I’m not going to be able to make these choices for you, and now you’ve taken on a very difficult choice, and I need to trust myself to trust you to make that choice.

On the same theme, another parent discussed the need for individualized care. “I think that it needs to be more individual, looking at the individual and respecting individuals is huge, and trusting that people know themselves.”

Observations of youth conducting online and other research, making informed decisions, and demonstrating consistency regarding their gender also informed parents’ perceptions that their children were capable decision-makers. Several parents commented on the thorough research undertaken by their children and even depended on their children to provide them with information. **“**Following her lead, and like I said, she’s very smart. She doesn’t do things just like that. She researches it. She looks into it. She asks questions. So, I was comfortable with it.” Other parents took a more active role in evaluating their child’s understanding of information relevant to hormone therapy decision-making. This parent had a strong understanding of informed consent processes through work in social services/healthcare. “I was able to say to her, ‘Okay, so you understand that these are the time frames and once you hit those time frames, there’s stuff that’s not reversible.’ And she totally understood.” Beyond acquired reassurances that their children were making informed decisions, parents’ confidence that their children knew, and would remain consistent in, their gender, was also part of their evaluation that youth were capable of making decisions about hormone therapy. “This is who [my son] is, and there is absolutely not a doubt in my mind that he would ever turn around and say, ‘No, you know what, I think I got this wrong.’”

#### Youth

Some youth shared that their healthcare providers did not adequately recognize their maturity and decision-making capacity. “The assessor didn’t ask it in a very respectful way. It was more condescending. Like, ‘Oh, you’re just a teenager. You’ll change your mind.’” Differential treatment of youth and adults was also highlighted by one participant who appreciated the caring intent of healthcare providers but not how it manifested in the provision of care.

I think it’s very easy for the doctors to see everything as you’re under eighteen, you’re a child … And I think that comes out of a place of caring, but I think it comes out of a place of very misguided caring.

Frustration was expressed that healthcare providers did not recognize that youth were capable of making their own decisions about hormone therapy.

If the youth’s asking for hormones from their doctor, they’ve probably thought about it already. It’s not just, like, oh, I’m impulsive and I’m going to go talk my doctor about hormones right now because I don’t know what I’m talking about. I think youth know what they want … They’ve thought about it enough that they know they want to proceed in the next steps.

They challenged the idea that any youth would pursue hormone therapy impulsively, even if youth do make some decisions without thinking them through. “I understand that I’m young, and I understand that a lot of young people are immature and can make rash decisions, but don’t colour us all the same shade of blue, because we’re not.”

Youth who researched or initiated hormone therapy at a younger age talked about their emerging capacity to make informed healthcare decisions. “I did all of the research, and it started to get more in-depth when I was maybe eleven or twelve and I started to sort of grow more mature.” One youth who started hormone therapy at a younger age discussed the importance of parental and healthcare provider support in making his decision.!

My mom was just really supportive and was like, “I’m here to support you,” and just helped me make the decision. I was really, really scared because I was worried that maybe I wasn’t ready, but [my mom and my healthcare provider] talked to me about it and helped me decide that’s what I wanted to do.

He had recognized that he was not ready to independently weigh the risks and benefits and had therefore requested support from trusted adults as his decision-making capacity developed.

While some youth described a process wherein a healthcare provider supported them to make their own decisions, others encountered challenges around healthcare providers requiring parental consent even when youth were deemed capable. One youth in government care, whose hormone therapy readiness assessor felt they were ready to start hormone therapy, described encountering challenges when meeting with the healthcare provider who was going to prescribe medication. “It was kind of a kerfuffle because I couldn’t start hormones on the exact day, because [that healthcare provider] needed consent from my parents.” Youth expressed preferences for healthcare provider approaches that were supportive of their capacity to make decisions and frustration with barriers to care that they perceived to be rooted in healthcare provider bias—specifically around youth capacity to consent and youth ability to know their gender—and a fear of litigation.

Several participants were asked about their decision-making styles and if these differed between hormone therapy decision-making and other decisions in their lives. Decision-making approaches included analysing possibilities and realistic outcomes, extensive deliberation, trusting instincts, weighing pros and cons, and discussing the decision with trusted people. Many youth described a consistent decision-making approach in their lives, for example, weighing the pros and cons surrounding initiating hormone therapy or choosing a post-secondary educational institution. Others contrasted their hormone therapy decision-making style with how they made less consequential decisions in their daily life. “This wasn’t a split-second decision like a lot of my decisions are. It actually had thought to it and took a while to decide. So, it was a very solid decision in my life.”

### Elements of Capacity

The data most relevant to analysis of whether trans youth can possess capacity to consent to hormone therapy are data from youth interviews. Youth descriptions of their hormone therapy decision-making processes are organized here around five key components of healthcare decision-making capacity evaluation: understanding of the proposed treatment, understanding of the anticipated effects (e.g., desired, side effects), understanding of alternatives to the proposed treatment, the ability to weigh the risks and benefits of reasonably foreseeable outcomes of various options, and making a decision that is consistent with one’s values (e.g., related to health goals, gender goals, life goals).

#### Understanding of Relevant Information

Youth acquired information about hormone therapy and its risks and benefits through independent online research and most had supplemented this with information gained through interaction with others (e.g., healthcare providers, parents, peers). They generally described having a solid understanding of the details, risks, and benefits of hormone therapy.

When I was talking to my endo[crinologist] and he handed me that packet of everything that would happen, I could have listed you those off the back of my hand. I literally knew everything that was on that packet beforehand—I think they kind of underestimated me in that sense.

There was evidence youth were seeking and triangulating information from a variety of sources to inform their decision-making, rather than relying on one source. For example, one youth said the hormone therapy-related questions she asked the healthcare provider “were the same ones that I’d asked the Internet. I just wanted to double-check them.” Another youth discussed accuracy of various sources and the need to verify information with a healthcare provider when it could not be triangulated online.

I’ll end up looking that up and kind of doing my own research on it, just to make sure that it’s actually correct … There are some people who kind of put false information out there … And once in a while I’ll ask the doctor: “Is this accurate? What more can you tell me about that?”

Though confident in their research, some youth felt reassured by healthcare provider validation of their readiness to start, and understanding of, hormone therapy.

My psychologist did agree that I knew what I was getting myself into more or less, and that she thought I was mature enough that she didn’t see any problem with me going on hormones, and that I was going into it informed.

Overall, as would be as expected, youth who were further through their decision-making process (i.e., had interacted with healthcare providers, had initiated hormone therapy) demonstrated a more comprehensive understanding of hormone therapy as a medical intervention.

#### Appreciation in the Context of One’s Own Life

Youth gave detailed and accurate descriptions of the anticipated effects of hormone therapy on their bodies—both desired outcomes and side effects. They accurately described anticipated outcomes, such as structural, body fat distribution, hair, voice, and emotional changes. The variability in effects of hormone therapy was also recognized. “I know that you can’t predict what does and doesn’t happen. Like, you can look at your family history to see what happened to other people, but at the end of the day there’s no guarantees.” Also evident was insight into the implications of long-term hormone therapy.

I made the decision that I wanted to be on hormones for the rest of my life or unless health problems that had to force me to be off them. And I know I might have to stop taking hormones before surgery.

Detailed descriptions of risks and how they were relevant to an individual based on their personal and family medical history were balanced against severe distress and depression related to not pursuing hormone therapy.

I think [the risk of heart disease] was the only thing that I’d worried about, and my mom and my dad had worried about, just because I’ve had my fair share of health problems. But also, I think they were more scared about what would happen if I didn’t go on them, and if I got back to that dark place, because I can’t remember how I got myself out of it.

The decision to initiate hormone therapy was not taken lightly. This youth, who had spent years deciding whether and when hormone therapy was the right option for him, stated,

I wanted to make sure, one hundred per cent … that this is what I need to do. I have to make that decision, and this is something that I’m going to have to do for the rest of my life … this is a big commitment, and I’m ok with making that commitment.

#### Reasoning About Treatment Options

Participants shared many thoughts about evaluating the pros and cons of treatment, their understanding of outcome variability, the need for support in weighing risks and benefits, and the long-term implications of hormone therapy. One youth stated succinctly, “I understand that there are side effects, but the possible reward is greater than those … They’re not going to kill me, so it’s all good.” Another youth compared the anticipated effects of hormone therapy to those of other medications when evaluating their level of risk.

I looked at all the possible side effects, or the risks. But you know, there’s a lot of other drugs and prescriptions [that] have a lot of the same side effects and it usually doesn’t happen … so there wasn’t like, this big risk … It was just sort of worries where you’re like, is this going to affect me or not?

As regret was an issue raised by some parents and healthcare providers, many youth participants were invited to comment on this. Youth responses focused on evaluating how significantly regret might impact their lives, not having experienced regret after starting hormone therapy, and experiencing regret about not having started the process earlier.

Most youth were clear that non-treatment was not an option. They needed hormone therapy and saw this as the only viable course of action.

This is very much something that I’m doing and that I feel comfortable doing, and I can’t imagine going back to being called “she, her,” and going back to my birth name. Like, the idea of it makes me want to cringe.

In the words of another participant, “I’m not going to *not* be on anything. That wouldn’t work out for me.”

Other discussion about alternatives centred around the lack of alternative medical treatments and the life-or-death nature of access to hormone therapy for some people. This youth acknowledged reasons why healthcare providers would be reticent to move forward with hormone therapy.

We’re kids. They want to make sure we’re safe … And I think if there were more studies on it, that would be a huge comfort blanket for doctors. But right now, this is the one [medical intervention] … there [are] no other options.

One alternative to hormone therapy that was discussed was suicide. This topic was not directly addressed through interview questions but was spontaneously brought up by some youth. A well-supported youth commented on the likely outcome if she had been unable to access hormone therapy.

If [my parents] straight up said you can’t transition, I probably wouldn’t be alive right now. I was in a really bad place before I transitioned, and I think seeing myself come out of that through becoming who I actually am has been so relieving to my family.

This response was affirming of both the youth’s decision to access hormone therapy and her parents’ decision to support her, as she reported there would have been no acceptable alternatives other than suicide.

#### Communicate a Choice That Is Consistent With Values

Consistently, youth were able to clearly communicate about their hormone therapy needs and how they aligned with their value of living in and expressing their affirmed gender. Some youth who were unable to access hormone therapy discussed how uncomfortable they felt physically and socially at the present time. For example, “I have never wanted boobs, I never did, and I never have, I never will … I hate [my period] … I hate it so much.” In the words of another youth, “I’m not comfortable at home. I’m not comfortable at school. I’m not comfortable talking to people on the street.” Others shared how hormone therapy was integral to their evolving ability to live authentically in their gender. “Once I figured out that I identified as a trans guy, I was like, well, I know this is the next step I want to take after socially transitioning, is going on hormones.”

Imagining the future with or without hormone therapy was a pivotal moment in one youth’s journey. “I imagined myself at forty-five, never having taken hormones—I’ve never imagined myself as that ever in my whole life. Not once have I thought that’s what I would look like or be living as.” When confronted with discouragement from others, youth were able to give voice to the ways in which their decision to initiate hormone therapy was consistent with their gender and their values over time. “And [my dad] goes: ‘What if you change your mind? What if you decide this isn’t what you want to do?’ And I said, ‘Dad, it’s been two and a half years, this is what I have to do.’” Youth described hormone therapy as an intervention that was consistent with their core values related to gender and well-being and expressed decisions that were consistently held over time.

## Discussion

These results bring three points into focus. First, trans youth in this study (ages fourteen through eighteen) demonstrated understandings and abilities characteristic of the capacity to make decisions about hormone therapy initiation. Second, youth, parents, and healthcare providers interviewed generally agreed that youth in this age range can possess the capacity to consent to this care and that it should be evaluated on an individual basis, consistent with provincial legislation. Third, support was expressed for youth access to hormone therapy via an informed consent model of care. Here we make two ethical arguments in favour of minor trans youth consent for hormone therapy. From a deontological stance, we argue that capable minor youth should have the right to make decisions about hormone therapy, then drawing on a consequentialist approach, we assert that because trans youth have the greatest interests in whether or not they initiate hormone therapy, they should be granted the legal authority to consent to this care.

### The Right to Decide

Starting with a deontological perspective, we argue that capable trans youth should be allowed to make their own decisions about hormone therapy based on the premise that all people have basic rights to choice, to self-determination, to bodily integrity, and to be treated as an end rather than a means to an end. Several child and youth human rights enshrined in the United Nations Convention on the Rights of the Child are delineated here to further support our stance (United Nations 1989). Canada is a signatory to this convention and these rights are strongly reflected in Canadian health policy.

First and second are the rights to *preserve identity* (article 8) and to *freedom of expression* (article thirteen). Youth have a right to have their gender respected and to be able to freely express their gender. As stated by a parent participant, this is about “trusting that people know themselves.” Third and fourth are the rights to *have views given weight in accordance with age and maturity* (article twelve) and for the *rights of legal guardians to be exercised in a manner consistent with evolving capacities* (article five). Youth talked about knowing what they needed, and parents discussed trusting their children to make decisions about their healthcare. Findings indicated that many youth were supported to exercise their decisional rights as they developed the capacity to do so. Healthcare providers also endorsed the idea that trans youth can have the maturity and reflexivity to make decisions about hormone therapy, and the empirical data in this paper support the assertion that youth capacity can evolve sufficiently in the fourteen- through eighteen-year age range to facilitate informed decision-making about hormone therapy initiation.

Fifth, youth have a right to the *highest attainable standard of health and to access healthcare services* (article twenty-four). In the words of one youth, “there [are] no other options” besides hormone therapy for achieving an acceptable standard of health. Failing to support youth rights to consent to their own healthcare can result in youth being denied access to medically necessary healthcare services. If parents were allowed to exercise parental rights in ways that overruled the healthcare decision of capable trans youth (and their healthcare providers), not only would youth lose access to needed healthcare services and potentially experience compromised health outcomes, these conditions might also allow trans youth to be treated as a means to their parents’ ends. For example, a parent who rejected their child’s gender could then be permitted to sacrifice their child’s health as a means to achieve the parent’s ends of futilely attempting to force their child to be cisgender.

The sixth article involves the right to *survival and development* (article six). Youth have a right to give direction to their own development. For trans youth, critical components of development can include social and medical steps to support life in their affirmed gender. It is evident from the literature that trans youth who are supported in their gender development have much lower rates of suicidality, while those whose development is compromised by a lack of family support and access to healthcare have troubling outcomes (Russell et al. 2018; Travers et al. 2012; Veale et al. 2015). As stated outright by one study participant, for some, the only acceptable alternative to accessing needed gender-affirming healthcare services is suicide. Denying trans youth the right to access hormone therapy would therefore clearly be an affront to their right to development and potentially their right to survival.

### Authority to Consent

Some may counter the deontological perspective we have presented here—that youth have the right to consent to hormone therapy—with the argument that parental rights should override youth rights due to cultural or societal norms. While it is true that parents are typically involved in healthcare decision-making for their children, and that they generally attend to their children’s best interests, parental legal rights to make healthcare decisions are not necessarily absolute (Goodlander and Berg 2011). For example, within Canada and the United States, governments will intervene when parents are not acting in the best interests of their child and instead placing that child at risk of serious, preventable harm. In the case of a capable minor youth, who is best positioned to decide what is in their best interests? Is it the youth, the parent(s), or a third party (e.g., healthcare provider, child protective services, judge)? As trans youth have to live in their bodies and interact within their communities, we assert that they have the most invested in decisions being made about access to gender-affirming hormone therapy. Thus, we build a consequentialist argument in support of trans youth authority to consent to their own healthcare, arguing that trans youth have by far the greatest interests in the outcomes of such decisions.

Best interests is a concept that is subjective and sometimes difficult to operationalize, as it is based on judgements about quality of life (Beauchamp and Childress 2013; Kopelman 2007; Rhodes and Holzman 2014). Malek (2009) has established a framework for understanding the best interests of young people in the context of paediatric healthcare, through analysis of documents that draw on three diverse perspectives: human rights, child development, and philosophy. This framework is comprehensive and well-reasoned in its approach to codifying the best interests of youth, specifically within healthcare paradigms. We build upon the thirteen interests of children^1^ identified by Malek (2009), highlighting eight as most relevant in the context of gender-affirming care: life; health and healthcare; protection from abuse and neglect; emotional development; expression and communication; identity; sense of self; and autonomy.

*Life:* It is in the best interests of trans youth to live a life of normal human length, including being supported in their gender such that risk of suicide is mitigated (Russell et al. 2018). *Health and healthcare:* minors should have access to needed medical care, including gender-affirming healthcare deemed appropriate by qualified healthcare providers (Coleman et al. 2012; de Vries et al. 2014; Olson, Forbes, and Beltzer 2011; Rosenthal 2014). *Protection from abuse and neglect:* Children should be protected from abuse and neglect, including abuse based on gender and neglect of gender-related medical needs (e.g., hormone therapy). *Emotional development:* Young people should be able to develop emotionally; for trans youth this includes being able to develop while living in the gender they know themselves to be and to be free of distress from being forced to live otherwise (Ehrensaft 2016; Olson, Durwood, DeMeules, and McLaughlin 2016; Russell et al. 2018). *Expression and communication:* Youth should be supported to freely express themselves, including to authentically express their gender through medical intervention. *Identity:* Youth should be supported in their identity, be connected to their cultures, and be protected from discrimination; for trans youth this includes being supported in their gender identity, connecting with trans cultures, and being protected from anti-trans bias and discriminatory practices in healthcare (Clark et al. 2018; Gridley et al. 2016; Russell et al. 2018). *Sense of self:* It is in the interests of youth to have a sense of self, to have self-worth, and to respect themselves—aims that can be furthered by trusting youth to know their own gender and supporting their gender goals. *Autonomy:* Youth should have the ability to influence the course of their lives. Autonomy for trans youth is an interest of fundamental importance, as no one other than the youth themselves can know their own gender and what, if any, medical interventions are needed to live comfortably in that gender (Ehrensaft 2016; Hidalgo et al. 2013).

Trans youth have strong interests in life, access to healthcare, protection from neglect of their needs, emotional development, gender expression and identity, sense of self, and autonomously influencing the course of their own lives. Not only do trans youth know more about their own gender than anyone else can, no one else has more invested in the decisions made about their gender in adolescence, given the immediate and long-term implications of such decisions on physical and psychosocial development and well-being. As one parent succinctly stated, “It’s her body. It’s her future.”

Research is clear regarding negative health outcomes for trans youth whose autonomy and access to healthcare are compromised, whose emotional development, identity and expression are restricted, and whose sense of self is undermined by unsupportive families, professionals, and societies (Clark et al. 2018; Russell et al. 2018; Travers et al. 2012). Lack of access to gender-affirming care in adolescence can carry not only immediate harms but also elevated risk of future harms, such as avoidable, invasive, and/or risky interventions in adulthood. The growing body of research on trans youth health outcomes provides robust support for affirming approaches and access to gender-affirming medical interventions (e.g., hormone therapy) when needed. This underscores the importance of supporting the interests of trans youth, including trusting them to know themselves and what they need to survive and thrive in the world.

### Informed Consent Model of Care

The informed consent model of care is based on the premise that trans individuals “are the only ones who are best positioned, in the context of their lived experience, to assess and judge beneficence” (Cavanaugh, Hopwood, and Lambert 2016, 1149). This model allows for a balanced approach wherein healthcare providers support autonomy through informed consent and elimination of required mental health assessment and diagnosis, provide benefits of accessible care, mitigate harms through screening for health concerns and connecting people with care and supports, and promote justice through interrupting pathologizing practices. As Cavanaugh et al. (2016) have stated, the informed consent model of care holds potential for practice with youth, but clinical uptake and research have been stymied by legal limitations. For example, access to the informed consent model is determined, in places such as the United States, by the legal age of consent for medical care rather than on empirical research concerning the capacity of youth to make healthcare decisions. Let us be clear that we strongly support ensuring mental healthcare is available to trans youth and their families when they are navigating challenging circumstances. We also contend that youth with decision-making capacity should be able to access gender-affirming care through the informed consent model—which ensures that appropriate mental health screening and supports are available—without requiring stigmatizing and potentially burdensome in-depth mental-health assessments.

Here we have presented evidence related to trans youth capacity to consent to hormone therapy and confirmation that healthcare providers are using the informed consent model of care with minor youth, in accordance with relevant legislation. We have based our deontological argument in favour of providing youth with the legal authority to consent to hormone therapy on the rights enshrined in the UN Convention of the Child, as these universal rights of children are supported by the vast majority of countries around the world. A major argument against youth rights to consent to gender-affirming care is that parents should have the right to make healthcare decisions for their children until the age of majority. However, as we have noted in our consequentialist argument, this is not an absolute right in places such as the United States, where parental rights can be overridden when the best interests of a child are in jeopardy. Application of Malek’s (2009) best interest framework has illustrated how supporting youth autonomy to consent to hormone therapy is justifiable in terms of best interests, given the empirical data regarding youth capacity to consent to this care.

Therefore, we find that granting youth with decisional capacity the legal authority to consent to gender-affirming hormone therapy to be ethically justifiable, while restricting their autonomy may violate their best interests and result in harmful outcomes. Within trans healthcare, the informed consent model is widely used with adults, to support autonomy, reduce harm, and promote justice for trans populations. Providing informed consent care for trans youth is consistent with the empirical findings and holds potential for expanding access to medically necessary gender-affirming care and supporting strong health outcomes for this population.

## Conclusion

Decisions made about access to gender-affirming care will impact an individual trans youth far more than any other stakeholder. A youth has the most to gain and the most to lose. While hormone therapy is not medically necessary for all trans youth, for some it is critical to well-being in both the short and long term. The empirical evidence presented indicates that youth aged fourteen through eighteen can demonstrate understandings and abilities characteristic of the capacity to consent to hormone therapy. The rights of minors and core interests related to determinations of best interests in paediatric healthcare have grounded our normative analysis and support the conclusion that it is trans youth who are best positioned to make decisions about their own gender healthcare. Finally, we argue that since the informed consent model of gender-affirming care has been developed to support self-determination of capable decision-makers, it would be appropriate to support capable youth to access this model of care and that denying access based on an arbitrary age of consent lacks ethical justification. Drawing on this foundation of empirical evidence, human rights, and best interests, we conclude that granting trans youth with decisional capacity both the right and the legal authority to consent to hormone therapy is ethically justified.

## Footnotes

1 The thirteen interests identified by Malek (2009) include life; health and healthcare; basic needs; protection from neglect and abuse; emotional development; play and pleasure; education and cognitive development; expression and communication; interaction; parental relationship; identity; sense of self; autonomy.
